# Supplementary material for: The PINK1 p.Asn521Thr Variant Is Associated with Earlier Disease Onset in GRN/C9orf72 Frontotemporal Lobar Degeneration
Source: Int J Mol Sci. 2022 Oct 25;23(21):12847. doi: 10.3390/ijms232112847 (PMC9656574; doi:10.3390/ijms232112847)
Supplement: Supplementary file 1 [file ijms-23-12847-s001.zip › ijms-1922450_Supplementary_Material_last.pdf]

Supplementary Materials

The *PINK1* Asn521Thr Variant Anticipates Disease Onset in *GRN*/*C9orf72* Frontotemporal Lobar Degeneration

Giacomina Rossi[1,†], Erika Salvi[2,†], Luisa Benussi[3], Elkadia Mehmeti[2], Andrea Geviti[4], Sonia Bellini[3], Antonio Longobardi[3], Alessandro Facconi[4], Matteo Carrara[4], Cristian Bonvicini[3], Roland Nicsanu[3], Claudia Saraceno[3], Martina Ricci[1], Giorgio Giaccone[1], Giuliano Binetti[5] and Roberta Ghidoni[3,*]

[1] Neurology V and Neuropathology Unit, Fondazione IRCCS Istituto Neurologico Carlo Besta, 20133 Milan, Italy
[2] Neuroalgology Unit, Fondazione IRCCS Istituto Neurologico Carlo Besta, 20133 Milan, Italy
[3] Molecular Markers Laboratory, IRCCS Istituto Centro San Giovanni di Dio Fatebenefratelli, 25125 Brescia, Italy
[4] Service of Statistics, IRCCS Istituto Centro San Giovanni di Dio Fatebenefratelli, 25125 Brescia, Italy
[5] MAC-Memory Clinic and Molecular Markers Laboratory, IRCCS Istituto Centro San Giovanni di Dio Fatebenefratelli, 25125 Brescia, Italy
* Correspondence: rghidoni@fatebenefratelli.eu, Tel.: 0039-030-3501725
† These authors contributed equally to this work.

Table S1: Low frequency and common genetic variants associated with age of onset with nominal p-value <0.05. The variants are ordered by linear p-value. rs1043424 survived FDR correction.

SNP	GENE	Location	c.pos	p.pos	P Linear	P Linear FDR	Beta Linear	CI (95%) Linear	P Logistic	P Logistic FDR	OR Logistic	CI (95%) Logistic
rs1043424	*PINK1*	missense	c.1562A>C	p.Asn521Thr	5.28E-04	0.044	-5.91	-9.13÷-2.68	0.042	1	2.38	1.03÷5.48
rs6674775	*LAMTOR5*	missense	c.322-35G>A		2.14E-02	1	3.23	0.52÷5.93	0.079	1	0.57	0.30÷1.07

C.pos, coding position; p.pos, protein position. P and Beta linear, p-value and beta coefficient derived from linear regression; P and OR Logistic, p-value and odds ratio derived from logistic regression.

Figure S1: Kaplan-Meier curve showing disease incidence in the three rs1043424 *PINK1* genotypes in *GRN* mutation carriers.

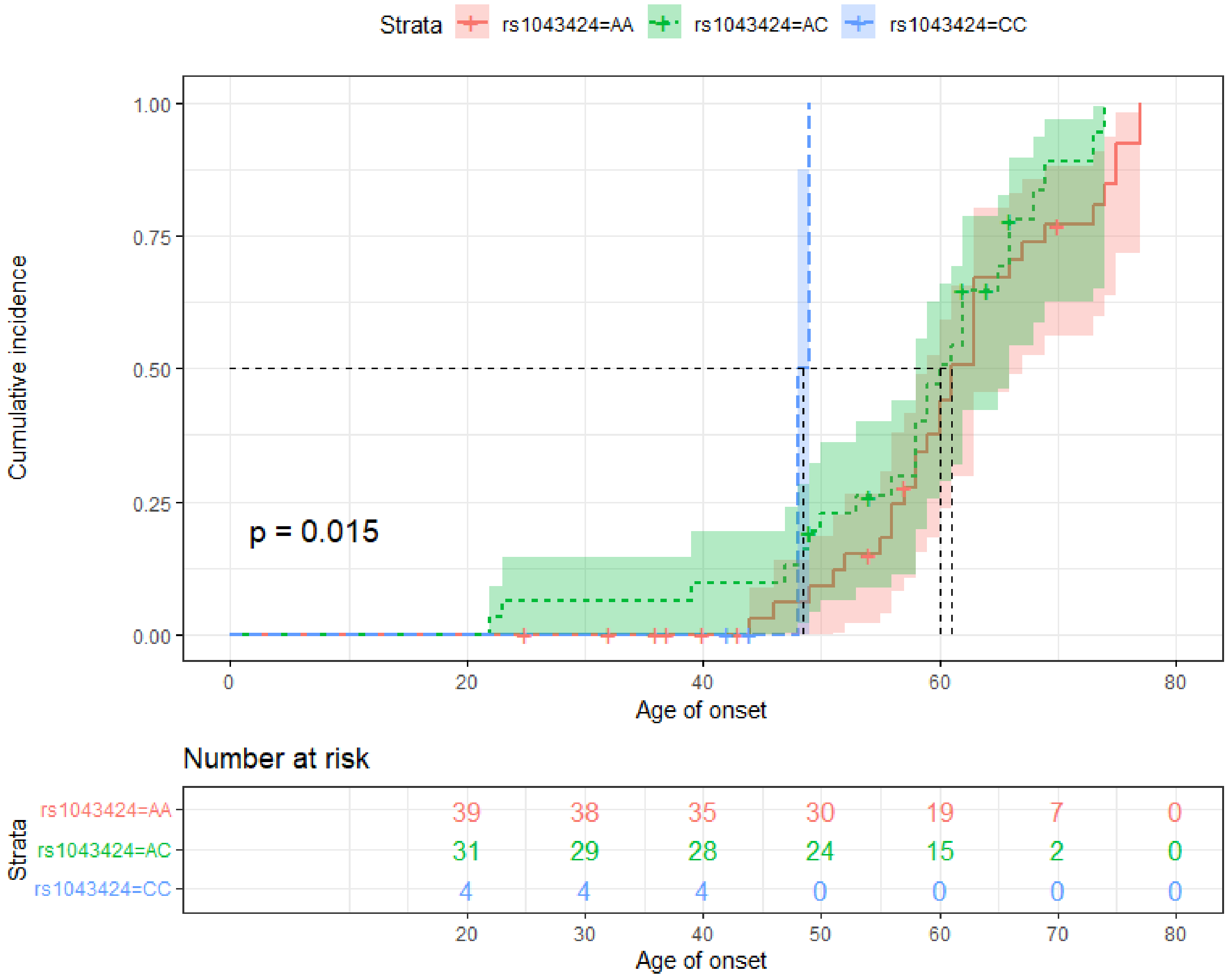

Figure S2: Kaplan-Meier curve showing disease incidence in the three rs1043424 *PINK1* genotypes in *C9orf72* mutation carriers.

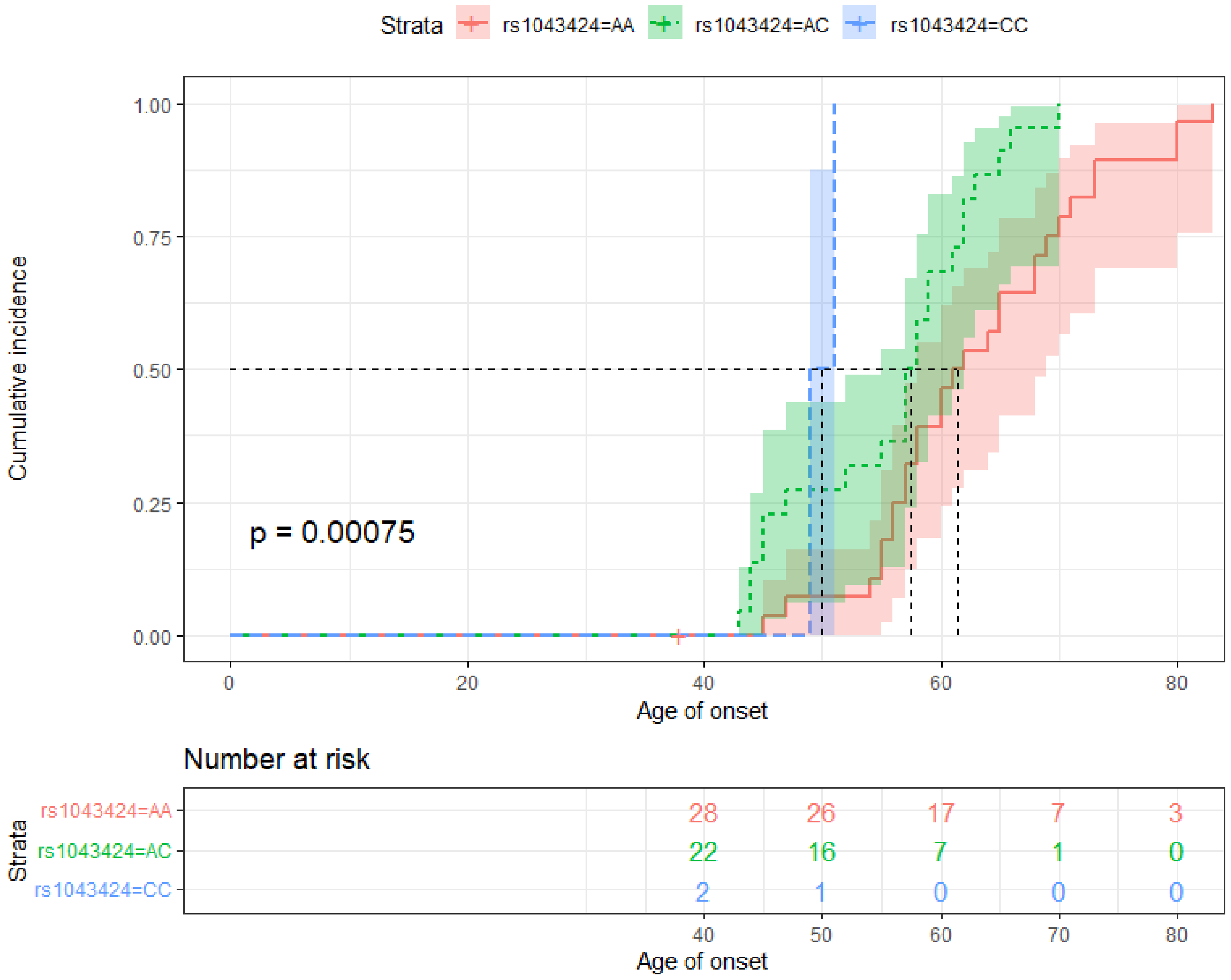

Table S2. Gene burden analysis.

Trait	GENE	CHR	Variants (N)	Singleton Variants (N)	P-skato	P-burden	OR
Early onset	*DPP7*	9	6	3	2.39E-01	1.73E-01	4.35
	RTN4	2	6	4	1.53E-01	1.97E-01	2.60
Late onset	*ATP13A2*	1	4	2	1.10E-01	9.73E-02	0.31
	ATP6V0A1	17	3	3	2.32E-01	2.04E-01	0.21
	DYNC1H1	14	6	3	2.80E-01	1.62E-01	0.44

CHR, chromosome; OR>1 indicates an excess of rare variants in early onset group whereas OR<1 in late onset patients.

Figure S3. Schematic representation of (**A**) *DPP7* and (**B**) *RTN4* structure and localization of rare genetic variants. Red dots indicate the variants exclusively carried by early-onset patients, light blue dots refer to variants identified also in late of onset samples and yellow dots represent variants exclusively carried by late of onset patients. Tables below the figures report the list of rare variants in each gene, with relative frequency in late and early onset patients. M, missense; CID, conservative inframe deletion; p.pos, protein position.

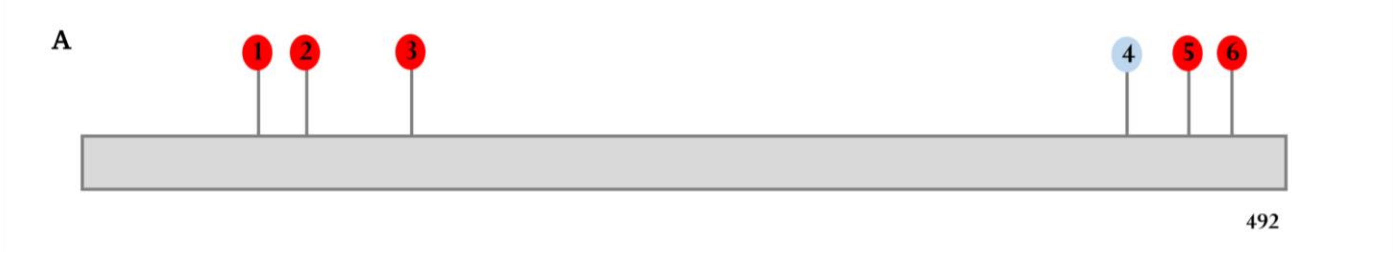

	GENE	SNPid	LOCATION	MAF	rs	Late	Early	p.pos
1	DPP7	9:140008816_C/T	M	0.005	rs759997556	0/0/30	0/1/68	p.Gly67Asp
2	DPP7	9:140008769_C/G	M	0.010	rs199586193	0/0/30	0/2/67	p.Ala83Pro
3	DPP7	9:140008443_G/A	M	0.015	rs150267769	0/0/30	0/3/66	p.Ser120Phe
4	DPP7	9:140005383_G/A	M	0.010	rs117203759	0/1/29	0/1/68	p.Ala431Val
5	DPP7	9:140005171_CGAT/C	CID	0.005	rs556718103	0/0/30	0/1/68	p.Ile469del
6	DPP7	9:140005147_G/A	M	0.005	rs144785203	0/0/30	0/1/68	p.Arg478Cys

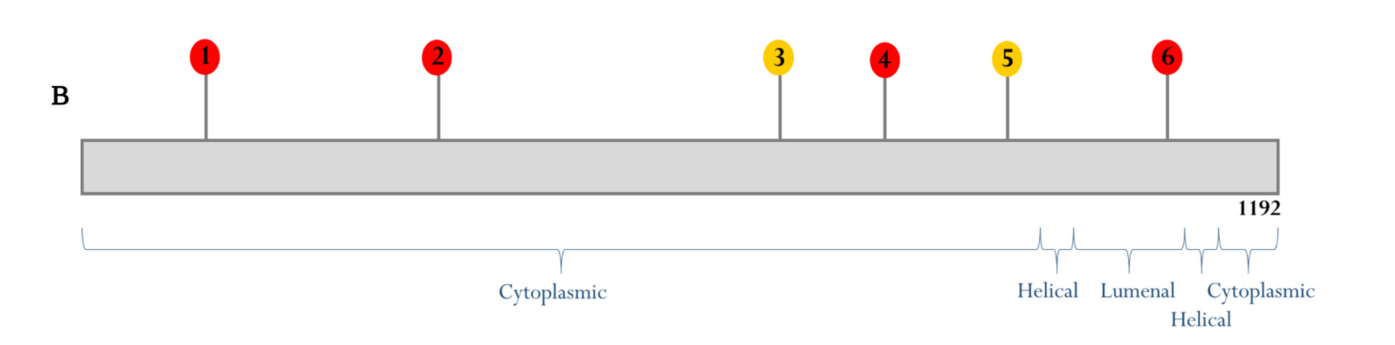

	GENE	SNPid	LOCATION	MAF	rs	Late	Early	p.pos
1	RTN4	2:55277165_C/T	M	0.012	rs201598949	0/0/25	1/0/55	p.Arg91Gln
2	RTN4	2:55254165_T/A	M	0.045	rs11677099	0/0/30	1/7/61	p.Asp357Val
3	RTN4	2:55253062_C/T	M	0.005	rs147845919	0/1/29	0/0/69	p.Glu725Lys
4	RTN4	2:55252764_T/C	M	0.005	rs201310203	0/0/30	0/1/68	p.Lys824Arg
5	RTN4	2:55252359_G/C	M	0.005	2:55252359	0/1/29	0/0/69	p.Ala959Gly
6	RTN4	2:55214639_C/G	M	0.005	rs11549898	0/0/30	0/1/68	p.Gly1070Ala
